# Immunological Responses Elicited by Different Infection Regimes with *Strongyloides ratti*

**DOI:** 10.1371/journal.pone.0002509

**Published:** 2008-06-25

**Authors:** Steve Paterson, Clare Wilkes, Colin Bleay, Mark E. Viney

**Affiliations:** 1 School of Biological Sciences, University of Liverpool, Liverpool, United Kingdom; 2 School of Biological Sciences, University of Bristol, Bristol, United Kingdom; Swiss Tropical Institute, Switzerland

## Abstract

Nematode infections are a ubiquitous feature of vertebrate life. In nature, such nematode infections are acquired by continued exposure to infective stages over a prolonged period of time. By contrast, experimental laboratory infections are typically induced by the administration of a single (and often large) dose of infective stages. Previous work has shown that the size of an infection dose can have significant effects on anti-nematode immune responses. Here we investigated the effect of different infection regimes of *Strongyloides ratti*, comparing single and repeated dose infections, on the host immune response that was elicited. We considered and compared infections of the same size, but administered in different ways. We considered infection size in two ways: the maximum dose of worms administered and the cumulative worm exposure time. We found that both infection regimes resulted in Th2-type immune response, characterised by IL4 and IL13 produced by *S. ratti* stimulated mesenteric lymph node cells, anti-*S. ratti* IgG_1_ and intestinal rat mast cell protease II (RMCPII) production. We observed some small quantitative immunological differences between different infection regimes, in which the concentration of IL4, IL13, anti-*S. ratti* IgG_1_ and IgG_2a_ and RMCPII were affected. However, these differences were quantitatively relatively modest compared with the temporal dynamics of the anti-*S. ratti* immune response as a whole.

## Introduction

Laboratory models of nematode infection are widely used to investigate how the vertebrate immune system responds to nematode infection and to then use this information to understand nematode infections, particularly of humans, domestic and wild animals [1]–[3]. A pervasive difficulty of using such laboratory models is that these models may not accurately model ‘natural’ infections. A consistent concern is that the way in which infections are initiated in the laboratory may cause artefacts in the host-parasite interaction [4]. Thus, for most geohelminth species, hosts in the field are continuously exposed to infective larvae or eggs, at low doses, over an extended period of time; so-called ‘trickle’ infections. By contrast, laboratory infections are most often initiated with single, large doses of infective stages. Therefore, a specific concern in the use of laboratory models to understand ‘natural’ infections, is whether infection regimes (*e.g.* multiple small doses compared with a single large dose) qualitatively and/or quantitatively affect the immune responses generated during these infections.

Different infection regimes have been most thoroughly investigated for *Nippostrongylus brasiliensis* infections of rats. Trickle infections of *N. brasiliensis* in the rat can establish long lived (*e.g.* 12 weeks ) infections [5] which contrasts with single dose infections which result in short lived (*e.g.* 3 weeks) infections [6]. However, formal comparison of single and trickle dose infections within one experiment has not, as far as we are aware, been made. Further, in some of these trickle-infection regimes, hosts were initially exposed to a large single dose infection [7], [8]. In other apparently long-lived trickle *N. brasiliensis* infections, the worm burdens late in infection may not have been larger in size than the residual population that remains at the ‘end’ of a single dose infection [7]. Other experiments have also suggested that host age affects the establishment of *N. brasiliensis* trickle infections [9], [10], and that trickle infection regimes may affect pre-intestinal, rather than intestinal, stages [11]. In summary, it is not fully clear whether or not (and if so, how) single and trickle dose initiated *N. brasiliensis* infections differ.

Immune responses elicited by, and to varying extents protective against, nematode infection are typically of a T helper 2 (Th2)-type, particularly characterised by production of the cytokines IL-4 and IL-13 [12]. Conversely, Th1-type responses are, comparatively, down-regulated during nematode infection. Experimental evidence for two nematode species shows that this Th1 - Th2 axis is affected by parasite dose. For *Trichuris muris* infections, Th1-type immune responses occurred in animals given repeated low dose infections, *i.e.* a trickle-style infection; latterly, the immune response developed into a protective Th2-type response [13]. In contrast, a single high dose infection resulted in a Th2-type response directly. In these experiments, the mode of delivery (*i.e.* trickle or single dose) or the size of the dose given could be responsible for the different immunological effects. For *Strongyloides ratti* infections in rats, the host immune response changes both qualitatively from a Th1- to a Th2-type immune response and the Th2-type response increases quantitatively with higher dose *S. ratti* infections [14].

Single, high-dose infections typical of experimental studies may be of limited relevance to understanding the biology of natural infections [15]. In the wild, most hosts have low worm burdens for most of their lives, which is likely to have shaped the evolution of the host immune response. For this reason, host immune responses to high does infections may be unusual. In low dose infections it could be envisaged that expending a lot of energy and resources to mount an immune response against a few worms, or to repair the resulting immunopathological damage, may be a poor strategy, especially since hosts will be continuously exposed and will rapidly reacquire infections. Therefore, it may be beneficial to a host to ‘accept’ some level of harm from low worm burdens, because the cost of preventing that harm outweighs the cost of the harm itself [16].

**Figure 1:**
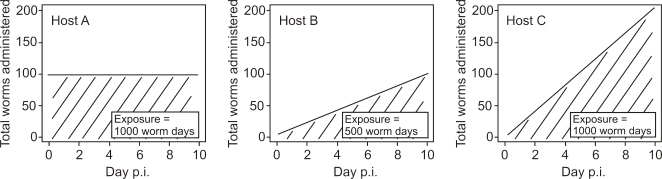
Diagrammatic illustration of infective dose measured as the number of worms received and as the worm exposure time. Host A receives 100 infective L3s (iL3s) in a single day, host B receives 10 iL3s every day for 10 days and host C receives 20 iL3s every day for 10 days. Therefore, hosts A and B both receive 100 worms but host A has twice the worm exposure time compared with host B. Hosts A and C both have the same worm exposure time (1000 worm days) but host C received twice the number of worms compared with host A.

In summary, there are differences in infection parameters between ‘natural’ and laboratory infections and, where investigated, nematode dose can affect host immune responses. We have therefore considered what aspect of ‘dose’ causes these effects. We suggest two possibilities. Firstly, dose could be the maximum number of worms (or the quantity of antigen) given to a host. Alternatively, dose could be the worm (or antigen) exposure time, *i.e.* the product of the number of worms (or quantity of antigen) given to a host and the time for which the host is exposed to these worms (or antigen). Both these measures of ‘dose’ will differ between single or trickle dose infection regimes. Thus, consider three hosts (Figure 1): host A receives 100 infective L3s (iL3s) in a single day, host B receives 10 iL3s every day for 10 days and host C receives 20 iL3s every day for 10 days. After ten days, therefore, hosts A and B will both have received a total of 100 worms but the worm exposure time for host A will be twice that of host B. By contrast, host C will have received a total of 200 worms (twice that of hosts A and B) but will have the same worm exposure time as host A.

Here we have used experimental laboratory infections of *Strongyloides ratti* in rats to (i) investigate the effects of single dose and trickle dose infection regimes on the host immune response, and (ii) examine the temporal dynamics of host immune responses to single dose and trickle dose infections.

## Materials and Methods

### Parasites and infections

*Strongyloides ratti* is a natural parasite of brown rats, *Rattus norvegicus*. Other *Strongyloides* species infect a range of mammalian and other vertebrate hosts, including humans [17]. Hosts become infected by penetration of the skin by iL3s present in the environment. Such larvae then migrate through the host, which for *S. ratti* occurs *via* the nasopharyngeal region [18], finally arriving in the small intestine, where the adult parasites mature and reproduce [19].

In the work presented here, the isofemale line ED321 Heterogonic was used throughout [20]. Previous work suggests that different isofemale lines of *S. ratti* do not elicit genotype-specific immune responses [21]. All infections were initiated by subcutaneous injection of iL3s. The overall design was to compare infection doses the equivalent of either 5 iL3s per day or 10 iL3s per day between two groups of rats receiving these infections either as (i) repeated dose (*i.e.* trickle) infections (‘Trickle 5’ and ‘Trickle 10’) or as (ii) single dose infections (‘Single 2.5’, ‘Single 5’ and ‘Single 10’). These three single dose infection regimes acted as control groups, because they received the same dose measured by (a) the maximum number of worms or (b) the worm exposure time as the two trickle infection regimes (Figure 1 and Table 1). To do this, the Trickle 5 group were administered with an equivalent of 5 iL3s per day by infection on days 0, 3, 7, 10, 14, 17, 21, 24, 28 and 31 post infection (p.i.) with 5, 15, 20, 15, 20, 15, 20, 15, 20, 15 iL3s, respectively. The Trickle 10 group was given an equivalent of 10 iL3s per day by infection with twice the number of iL3s as the Trickle 5 group. Pairs of animals were sampled (see below) on days 14, 21, 28, 35 and 42 p.i. The control single dose infected animals were sampled at the same time points as these two trickle groups. Pairs of control single dose infected animals were administered with a single dose of iL3s on day 0 p.i. either half, equal to or double (Single 2.5, 5 and 10, respectively) the appropriate cumulative number of larvae that the contemporaneously sampled trickle infection animals had received. Thus, the Single 2.5 group received 27, 45, 62, 80 or 80; the Single 5 group received 55, 90, 125, 160 or 160; the Single 10 group received 110, 180, 250, 320 or 320 iL3s for animal sampled on days 14, 21, 28, 35 and 42 p.i., respectively (Table 1). To execute this experimental design, 100 female Wistar rats (100–200 g) (Harlan, UK) were split into two replicate blocks and into 5 groups of 10 rats within each block. Therefore, for each sample day for each of the 5 infection regimes there were four animals, with two within each replicate block of the experiment.

**Table 1:** Infection regime showing number of worms (N) and cumulative worm exposure time (Exposure) in worm days in each of the two trickle and three control single dose infection regimes at the five culling points at which immunological measures were taken.

Group	Time of cull (dpi)	Infection regime	N	Exposure
Single 2.5	15	27 iL3s on day 0 p.i.	27	270
	22	45 iL3s on day 0 p.i.	45	765
	29	62 iL3s on day 0 p.i.	62	1488
	36	80 iL3s on day 0 p.i.	80	2480
	43	80 iL3s on day 0 p.i.	80	3040
Trickle 5	15	5 iL3s *per* day*	55	275
	22	5 iL3s *per* day*	90	765
	29	5 iL3s *per* day*	125	1500
	36	5 iL3s *per* day*	160	2480
	43	5 iL3s *per* day*	160	2800
Single 5	15	55 iL3s on day 0 p.i.	55	550
	22	90 iL3s on day 0 p.i.	90	1530
	29	125 iL3s on day 0 p.i.	125	3000
	36	160 iL3s on day 0 p.i.	160	4960
	43	160 iL3s on day 0 p.i.	160	6080
Trickle 10	15	10 iL3s *per* day*	110	550
	22	10 iL3s *per* day*	180	1530
	29	10 iL3s *per* day*	250	3000
	36	10 iL3s *per* day*	320	4960
	43	10 iL3s *per* day*	320	5600
Single 10	15	110 iL3s on day 0 p.i.	110	1100
	22	180 iL3s on day 0 p.i.	180	3060
	29	250 iL3s on day 0 p.i.	250	6000
	36	320 iL3s on day 0 p.i.	320	9920
	43	320 iL3s on day 0 p.i.	320	12160

N and exposure are calculated assuming that the development of *S. ratti* adults takes 5 days following infection. Exposure is calculated as N x (Time of cull - 5) for single dose regimes and as ½ x N x (Time of cull - 5) for trickle dose regimes.

* These infection regimes are the daily equivalent of iL3s administered from day 0 p.i. to (Time of cull - 5) inclusive, as detailed in the methods.

Animals were sampled for parasitological and immunological analyses. To do this, for each animal, faeces were collected on the days p.i. indicated (above). The faeces were cultured and the number of larvae that developed was determined, as previously described [22], [23]. This is a measure of the viable reproductive output of an infection [22]. On the day following collection of faeces (*i.e.* days 15, 22, 29, 36 and 43, Table 1), animals were culled and the small intestine, spleen, mesenteric lymph nodes (MLN) and blood removed. The intestine was frozen; this was subsequently used to determine the number of parasitic females present in the small intestine, as previously described [22]. Combined, the reproductive output of the infection and the number of intestinal parasitic females was used to determine the *per capita* fecundity, as previously described [22]. Remaining intestinal material, serum, spleen and MLNs were processed for immunological measurement as previously described [14], [23]. Briefly, this was the concentration, measured by ELISA, of IL4, IL13 and IFNγ produced by splenocytes and MLNs stimulated *in vitro* with *S. ratti* parasite antigen (above the background concentration of the media-only controls). The concentration of anti-*S. ratti* IgG_1_, IgG_2a_ and IgG_2b_ and total IgE in serum and of of anti-*S. ratti* IgA and of rat mast cell protease II (RMCPII) in intestinal tissue were also measured by ELISA [23]. These measures were used because they have previously been shown to be significantly altered during primary *S. ratti* infections of rats [14], [23]. One animal died during the experiment, for causes apparently unrelated to the infection regime. Technical problems prevented accurate determination of the concentration of the cytokines in 10 samples, and these data were removed from further analyses.

### Statistical analysis

We sought to normalise the data for each of the immune parameters using a Box-Cox transformation [24] (Equation 1), to use these data in statistical tests of significance that assume a normal distribution.
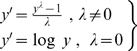
(1)Maximum likelihood was used to find a value of λ that resulted in the residuals for a linear model of each parameter regressed onto days p.i. (dpi) and dpi^2^ conforming as closely as possible to a normal distribution [25]. These residuals were then checked by eye using quantile-quantile plots. Values of λ found were 0.343 for MLN IL4, 0.343 for MLN IL13, 0.181 for MLN IFNγ, 0.0202 for spleen IFNγ, 0.0202 for IgE, 0.0202 for IgG_1_, and -0.101 for RMCPII. Transformed data for each parameter were then scaled to have a mean of zero and variance of 1. For spleen IL4, spleen IL13, IgA, IgG_2a_ and IgG_2b_, maximum likelihood estimates of λ failed to converge and/or quantile-quantile plots indicated that no value of λ provided a suitable transformation. For these measures, a binary response corresponding to presence/absence was used, where presence was defined as a detectable level of immunoglobulin isotype or cytokine (relative to corresponding negative control reactions). For analyses of immune parameters as a response variable, linear models were used for immune parameters that could be normalised, and generalised linear models (GLMs) with a binomial error structure were used for parameters as presence/absence data. For analyses of intestinal worm burden and *per capita* fecundity as response variables, GLMs with a negative binomial error structure were used. Principal component analysis [25] was conducted on the scaled parameters, MLN IL4, MLN IL13, MLN IFNγ, IgE, IgG_1_ and RMCPII. All analyses were performed in R v2.5.0 (www.r-project.org).

## Results

### Response of worm burden to infection regime and to immune parameters

**Figure 2:**
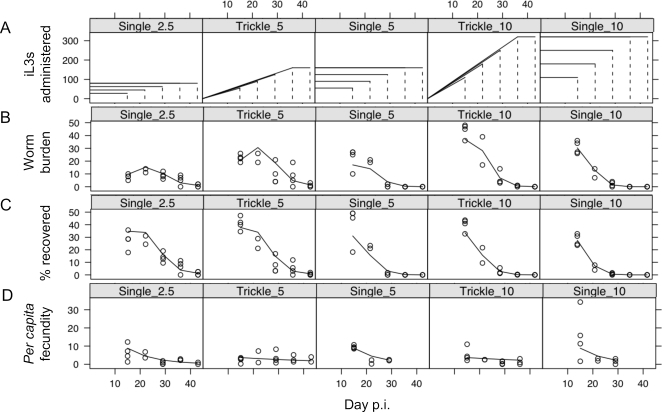
(A) Diagrammatic representation of the three control single dose, and two trickle dose infection regimes shown as the number of iL3s administered, which were sampled at days 14, 21, 28, 35 and 42 p.i., shown as vertical dotted lines; (B) the intestinal worm burden through time, and (C) this expressed as a percentage of the number of iL3s administered; and (D) the *per capita* fecundity of parasitic females. For (B–D) each point represents data from one animal and lines the best-fit predictions from minimal models.

The intestinal worm burden (*i.e.* the number of parasitic females recovered from a host) of all 5 infection regimes through time is shown in Figure 2. In all groups, the intestinal worm burden decreased during infection (Table 2) and this decrease was more rapid in the groups receiving more infective larvae (log(number of worms):dpi^2^, p<0.001, Table 2). This was consistent with our previous observation of density-dependent effects on *S. ratti* infections [14], [22]. As infections progressed, trickle dose infections resulted in progressively higher worm burdens compared with single dose infections (trickle:dpi^2^, p<0.001, Table 2). This is expected even in animals receiving the same number of worms but by different regimes, since in single dose infections the worm burden is only those worms that have survived from day 0 p.i., whereas in trickle dose infections the worm burden includes more recently acquired worms. Intestinal worm burden was significantly, and negatively, associated with both the concentration of IL4 and IL13 produced by the MLN cells stimulated with parasite antigen (Tables 3 and 4), but not with any other measured immune parameters. There was no difference between the single and trickle infection regimes in this negative association between cytokine concentration and intestinal worm burden (Tables 3 and 4).

**Table 2:** The results of generalized linear models of intestinal worm burden through time (dpi) in trickle and single dose infection regimes.

Term	Coefficient (±s.e.)	p-value
Intercept	−3.684±1.140	
log(number of worms*)	0.657±0.267	0.016
trickle	−0.308±0.277	0.249
dpi^2^	0.014±0.002	<0.001
log(number of worms*): dpi^2^	−0.0037±0.0005	<0.001
trickle:dpi^2^	0.0022±0.0004	<0.001

AIC = 420.7, k = 5.24±1.74, residual df = 83.

* - number of iL3s administered

**Table 3:** The association between intestinal worm burden and the concentration of IL4 produced by MLN stimulated with parasite antigen.

Term	Coefficient	p-value
Intercept	−3.408±1.108	
trickle	−0.379±0.226	0.10
dpi	−0.085±0.052	0.12
log(number of worms*)	0.838±0.254	<0.01
MLN IL4	0.362±0.199	0.08
dpi^2^	0.016±0.002	
dpi:MLN IL4	−0.024±0.008	<0.01
trickle:dpi^2^	0.0023±0.0004	<0.001
log(number of worms*):dpi^2^	−0.0036±0.0004	<0.001

AIC = 408.9, k = 9.50±3.89, residual df = 80.

* - number of iL3s administered

**Table 4:** The association between intestinal worm burden and the concentration of IL13 produced by MLN stimulated with parasite antigen.

Term	Coefficient	p-value
Intercept	−5.024±1.196	
trickle	−0.456±0.264	0.08
log(number of worms*)	0.982±0.282	<0.001
MLN IL13	−0.200±0.075	<0.01
dpi^2^	0.016±0.002	
trickle:dpi^2^	0.0024±0.0004	<0.001
log(number of worms*):dpi^2^	−0.0040±0.0005	<0.001

AIC = 415.8, k = 6.18±2.12, residual df = 82.

* - number of iL3s administered

### Response of per capita fecundity to infection regime and to immune parameters

*Per capita* fecundity of parasitic females decreased during infection in all of the three control single dose infection regimes but appeared to remain relatively constant through time in the trickle dose infections (Figure 2) (trickle:dpi, p<0.01, Table 5). *Per capita* fecundity was significantly, and negatively, associated with the concentration of IL13 produced by MLN cells stimulated with parasite antigen and with intestinal RMCPII concentration, but not with any other immune parameters (Table 6 and 7). Therefore, the concentration of IL13 produced by MLN was associated with two aspects of fitness, parasitic female survival and fecundity. In contrast, the concentration of IL4 produced by MLN and the intestinal concentration of RMCPII were separately associated with parasite survival and fecundity, respectively.

**Table 5:** The results of generalized linear models of *per capita* fecundity through time (dpi) in comparing trickle and single dose infection regimes.

Term	Coefficient (±s.e.)	p-value
Intercept	3.627±0.460	
trickle	−1.980±0.679	<0.01
dpi	−0.0968±0.0190	<0.01
trickle:dpi	0.0734±0.0265	<0.01

AIC = 536.8, k = 1.44±0.28, residual df = 56.

**Table 6:** The association between *per capita* fecundity and the concentration of IL13 produced by MLN stimulated with parasite antigen.

Term	Coefficient	p-value
Intercept	3.682±0.455	
trickle	−1.821±0.666	<0.01
dpi	0.100±0.018	
MLN IL13	0.793±0.383	0.07
trickle:dpi	0.070±0.026	<0.01
dpi:MLN IL13	−0.035±0.015	0.044

AIC = 536.4, k = 1.57±0.31, residual df = 54.

**Table 7:** The association between *per capita* fecundity and the concentration of RMCPII in intestinal tissue.

Term	Coefficient	p-value
Intercept	6.424±1.20	
dpi	−0.356±0.094	<0.01
dpi^2^	0.006±0.002	<0.01
RMCPII	1.140±0.355	<0.01
dpi:RMCPII	−0.045±0.015	<0.01

AIC = 532.4, k 1.61±0.32, residual df = 54

### Response of immune parameters to infection regime

**Figure 3:**
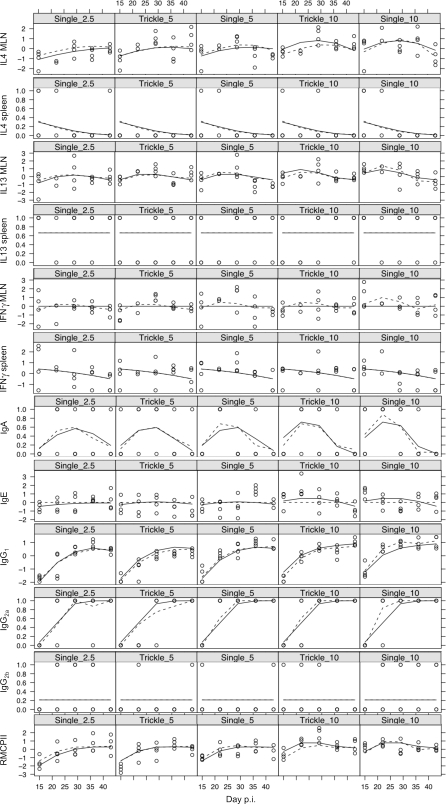
The temporal dynamics of 12 immune parameters in the three control single dose, and two trickle dose infection regimes at days 15, 22, 29, 36 and 43 p.i. The concentration of IL4 and IL13 produced by splenocytes stimulated with parasite antigen and the concentration of anti-*S. ratti* IgA, IgG2_a_ and IgG_2b_ are shown as presence/absence data. All other immune parameters have been transformed and scaled to have a mean of zero and variance of 1. Each point represents data from one animal. Best-fit predictions from minimal models are shown for explanatory variables of number of worms administered (solid lines) or worm exposure time (dotted lines).

We compared the temporal dynamics of the 12 measured immune parameters between the two infection regimes (Figure 3). This shows that for both infection regimes there were convex relationships with time p.i. for the concentration of IL4 and IL13 produced by MLN cells stimulated with parasite antigen and for the concentration of anti-*S. ratti* IgA in intestinal tissue. There was an asymptotic relationship for IgG_1_ and IgG_2a_ concentrations and for the concentration of RMCPII in intestinal tissue. For the remaining immune parameters measured there was no change in their concentration during the infections. Several of the immune measures increased with dose, where dose was measured as either (a) number of worms or (b) worm exposure time (Figure 1). Each of these two measures were tested in separate sets of models. For dose measured as number of worms, significant and positive associations were found with MLN IL4 (p<0.001), MLN IL13 (p<0.05), IgA (p<0.05), IgE (p<0.01), IgG_1_ (p<0.01) and RMCPII (p<0.01). For dose measured as worm exposure time, significant and positive associations were found with MLN IL4 (p<0.001), MLN IL13 (p<0.01), MLN IFNγ (p<0.05), IgA (p<0.01), IgG_1_ (p<0.001) and RMCPII (p<0.001). We then tested formally which, if either, of these two measures of dose better explained the observed variation in the measured immune parameters. This showed that the number of worms was a better explanatory variable for the concentration of IL4 produced by MLNs stimulated with parasite antigen and the concentration of RMCPII; in contrast, worm exposure time was a better explanatory variable for the concentration of IL13 produced by MLN stimulated with parasite antigen and the concentration of anti-*S. ratti* IgG_1_ and IgG_2a_ (Table 8). However, comparison of the predicted fits for each of these models to the observed data (Figure 3) shows only small differences in the fit of each model. Overall, this therefore suggests that, although parasite dose had a significant effect on the immune response, there were no biologically significant differences according to whether dose was measured as the number of worms received or as the worm exposure time.

**Table 8:** AIC for minimal models containing dose measured as (i) the number of worms received and (ii) the worm exposure time, as explanatory variables for the measured immune parameters.

Immune parameter	Number of worms*	Worm exposure time*
MLN IL4	**238.8**	241.4
Spleen IL4	60.8	60.3
MLN IL13	248.4	**246.2**
Spleen IL13	115.8	115.8
MLN IFNγ	255.6	255.7
Spleen IFNγ	247.5	247.5
IgA	115.9	114.3
IgE	282.1	283.9
IgG1	147.3	**123.6**
IgG2a	53.3	**47.8**
IgG2b	104.3	104.3
RMCPII	**232.0**	245.7

* Smaller AIC values indicate a more parsimonious model. Shown in bold is where minimal models for number of worms versus worm exposure time differ by at least 2 AIC units.

### Correlations among immune parameters

**Figure 4:**
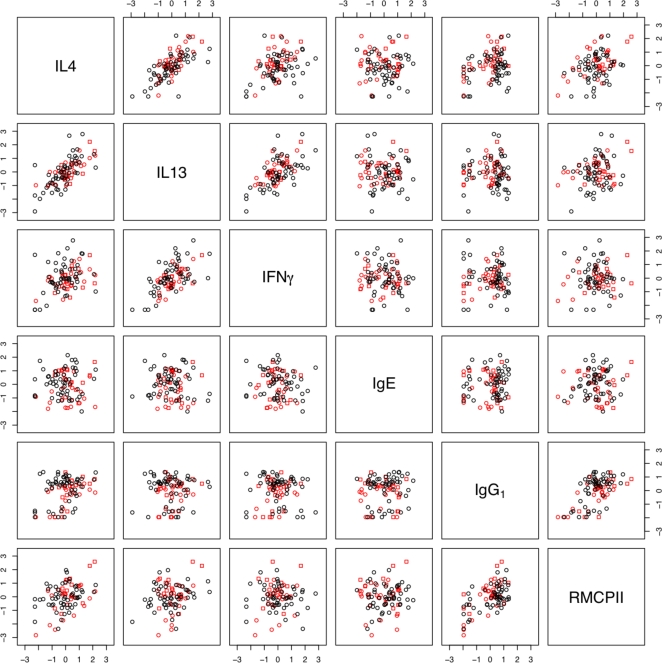
Correlations between the concentration of IL4, IL13, IFNγ produced by MLN stimulated by parasite antigen, anti-*S. ratti* IgG_1_, total IgE and RMCPII. Each point represents data from one animal. Black triangles (▵) are the Single 2.5 group, black circles (○) the Single 5 group, black squares the Single 10 group (□), red circles the Trickle 5 group and red squares the Trickle 10 group.

**Figure 5:**
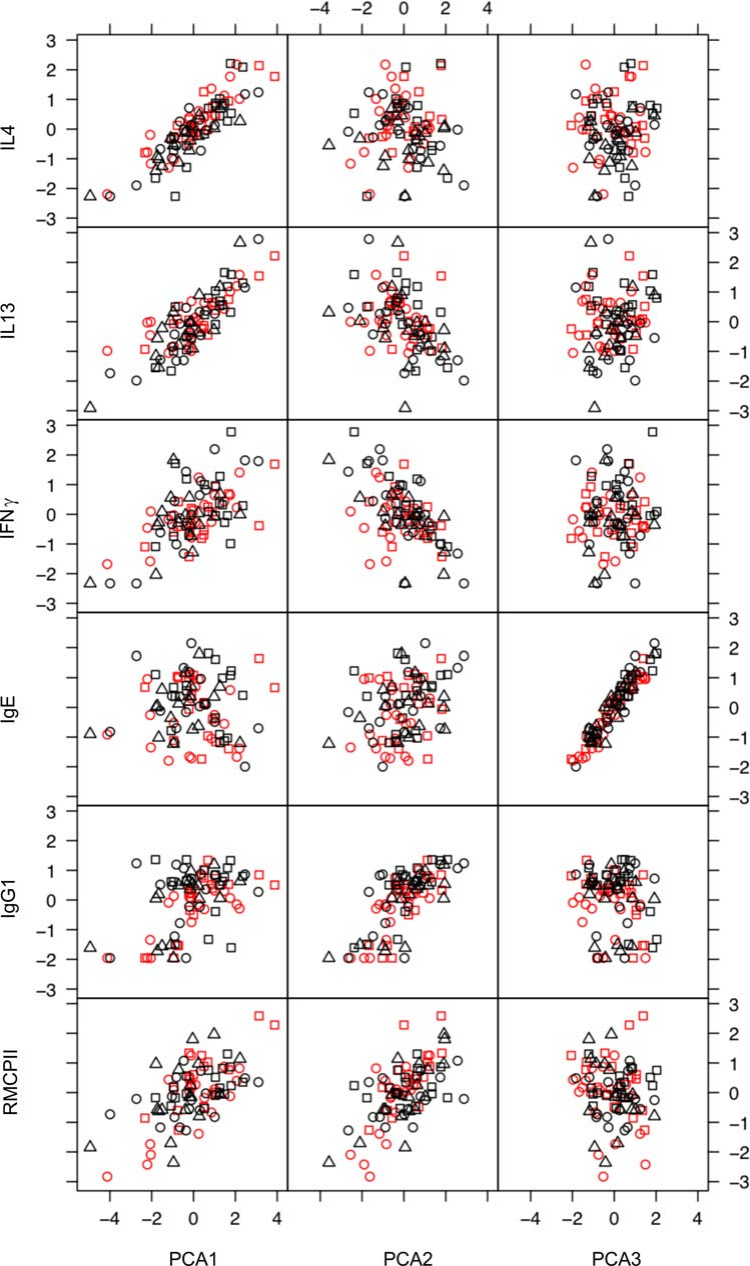
The concentrations of IL4, IL13, IFNγ produced by MLN stimulated by parasite antigen, anti-*S. ratti* IgG_1_, total IgE and RMCPII mapped onto principal components 1, 2 and 3. Black triangles (▵) are the Single 2.5 group, black circles (○) the Single 5 group, black squares the Single 10 group (□), red circles the Trickle 5 group and red squares the Trickle 10 group. Each point represents data from one animal.

**Figure 6:**
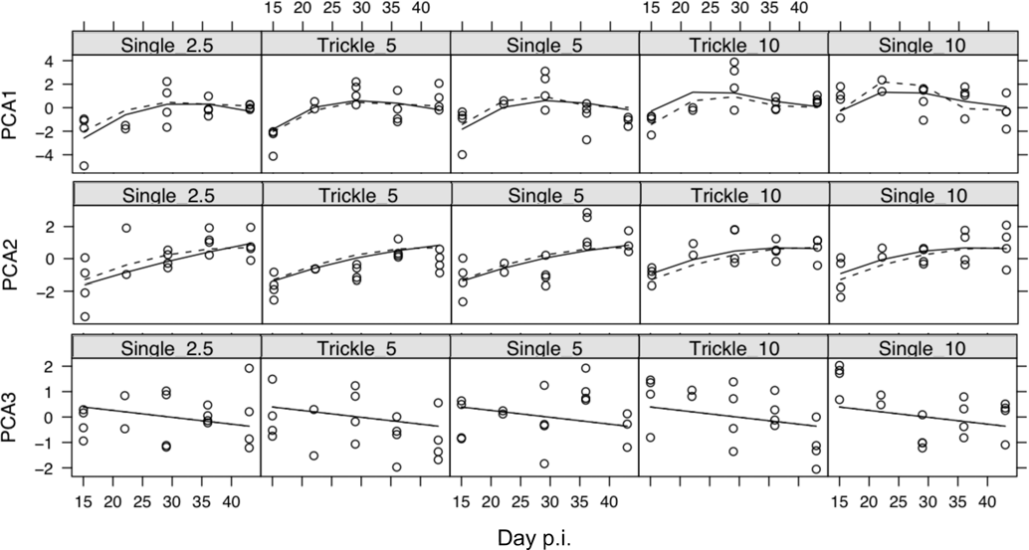
The temporal dynamics of principal component axes 1, 2, and 3 in the three control single dose, and two trickle dose infection regimes at sample days 15, 22, 29, 36 and 43 p.i. Each point represents data from one animal. Best-fit predictions from minimal models are shown for explanatory variables of number of worms administered (solid lines) or worm exposure time (dotted lines).

Since the immune parameters measured here interact with each other, we used principal component analysis to examine, first, the correlations among immune parameters and, second, whether the concerted responses of groups of immune parameters differed between infection regimes. This analysis was limited to parameters that could be statistically normalised, namely; the concentration of IL4, IL13 and IFNγ produced by MLN stimulated with parasite antigen, the serum concentration of total IgE, anti-*S. ratti* IgG_1_, and the concentration of RMCPII in intestinal tissue (Table 9 and Figure 4). This showed that the concentrations of IL4 and IL13 were positively correlated with each other and, to a lesser extent, with the concentration of IFNγ. The concentrations of IgG_1_ and RMCPII were positively correlated with each other. The data from trickle and single infections did not cluster into separate groups (Figure 4). This indicates (i) that there were no qualitative differences between the immune responses of the trickle and single dose infection regimes and (ii) that correlations between immune measures were not due to systematic differences between the immune responses of these different infection regimes. The concentration of IgE was uncorrelated to any of the other five immune parameters (Table 9). Principal component axis 1 (PCA1) corresponds to a positive correlation between all the immune measures except IgE, and appeared particularly strongly correlated with IL4 and IL13 (Figure 5). PCA1 increased asymptotically through time in all groups and was comparatively higher in high dose infections (Figure 6). PCA2 identified two groups of measures; (i) IL4, IL13 and IFNγ and (ii) IgE, IgG_1_ and RMCPII, where positive correlations were seen among measures within each group and negative correlations between groups (Table 10). IgE, which was only weakly correlated to any of the other immune parameters, was correlated closely with PCA3. PCA3 decreased during infection, with no apparent differences between infection regimes or infection doses. Taken together, PCA1, 2 and 3 describe 81% of the variance among IL4, IL13, IFNγ, IgE, IgG_1_ and RMCPII (Table 11).

**Table 9:** Pearson correlation coefficients between the concentrations of six immune parameters.

	MLN IL13	MLN IFNγ	IgE	IgG_1_	RMCPII
MLN IL4	0.71	0.37	0.01	0.34	0.40
MLN IL13		0.61	−0.05	0.03	0.24
MLN IFNγ			−0.09	0.00	0.04
IgE				0.06	0.06
IgG_1_					0.59
RMCPII					

**Table 10:** Principal component analysis of immune parameters showing the loadings of the measured immune parameters onto each principal component axis (PCA1-6).

	PCA1	PCA2	PCA3	PCA4	PCA5	PCA6
MLN IL4	0.558			0.489	0.400	−0.534
MLN IL13	0.530	−0.352		0.244		0.712
MLN IFNγ	0.390	−0.460		−0.738		−0.287
IgE		0.246	0.966			
IgG_1_	0.315	0.589		−0.387	0.523	0.312
RMCPII	0.394	0.507		−0.738		

Data are shown for loadings >0.2 and <−0.2

**Table 11:** Principal component analysis of immune parameters showing the proportion of variation explained by each principal component axis (PCA1-6).

	PCA1	PCA2	PCA3	PCA4	PCA5	PCA6
Standard deviation	1.54	1.21	0.98	0.74	0.64	0.42
Proportion of Variance	0.40	0.25	0.16	0.09	0.07	0.03
Cumulative Proportion	0.40	0.65	0.81	0.90	0.97	1.00

## Discussion

There are substantial differences between how nematode infections are naturally acquired by hosts and how nematode infections are initiated in the laboratory, characterised by trickle and single dose infection regimes, respectively. Previously, we have shown that the dose of *S. ratti* infections affects the host immune response, such that there was a Th1-type immune response for doses of 30 or fewer iL3s; at higher doses there was a Th2-type immune response [14]. Here we compared the immune responses of *S. ratti* infections initiated by single or trickle dose infection regimes, over a range of doses. While all animals received a maximum dose of more than 30 iL3s, the Trickle 5 group always received fewer than 30 iL3s on any single occasion. Nevertheless, for both single and trickle dose infection regimes across different sized doses a Th2-type immune response predominated. Neither did we find any substantial, quantitative immunological differences between these two infection regimes.

Consistent with previous findings, our experiments showed that the dose of worms administered, irrespective of the infection regime, was positively correlated with the concentration of IL4 and IL13 produced by parasite antigen-stimulated MLNs, the concentration of anti-*S. ratti* IgG_1_, IgA and total IgE, and RMCPII (Figure 3). However, we found that some of these immune parameters differed quantitatively between different measures of parasite dose. Thus, the concentration of IL4 produced by MLNs stimulated with parasite antigen and the concentration of RMCPII, correlated more closely with the maximum dose of worms administered; the concentration of IL13 produced by MLNs stimulated with parasite antigen and the concentration of anti-*S. ratti* IgG_1_ and IgG_2a_, correlated more closely with the cumulative worm exposure time. However, while these were statistically demonstrable differences their magnitude was small, especially compared to the overall dynamics of these immune parameters during an infection (Figure 3) [14]. In conclusion, our results indicate that the mode of delivery of an infection, whether by single or trickle dose infection regimes, has rather little effect on the host anti-*S. ratti* immune response. While this is a ‘negative’ result, it does suggest that within the parameters considered, that laboratory *S*. *ratti* infections may indeed model naturally acquired infections. The caveat to this conclusion is that other trickle infection regimes with different temporal schedules and periods may generate different results.

Intestinal worm burden differed markedly between treatment groups, with maximum worm burden appearing to occur earlier in higher dose infections (Figure 2b), consistent with the peak-shift commonly observed in age-intensity relationships of naturally-acquired nematode infections [26] and in experimental infections of *Heligmosomoides bakeri* [27]. The *per capita* fecundity of parasitic females in infections initiated with a single dose reduced during infection. This is consistent with our previous observation of immune-dependent density-dependent effects on *S. ratti* infections [22]. By contrast, the *per capita* fecundity of parasitic females in infections initiated with a trickle infection regime remained relatively constant during an infection. These infections will be similar to natural infections, in that the population of worms within a host is asynchronous. Thus, the population of worms within a host will contain both females being damaged by the host immune response and reducing their fecundity [22], [23], together with newly arrived parasitic females, not yet subject to these effects. For this reason, the average *per capita* fecundity of parasitic females in hosts infected (naturally or by artificial trickle infection regimes) in this way, will remain relatively constant through time.

Our immunological results are consistent with previous findings from experimental and field infections. Here we found that the concentration of IL4 late in an infection and of IL-13 throughout an infection, both produced by MLN, were associated with reduced worm burdens (Tables 3 and 4); and that, late in an infection the concentration of IL13 produced by the MLN and of RMCPII were associated with reduced *per capita* fecundity (Tables 6 and 7). Therefore, the concentration of IL13 produced by MLN was associated with two aspects of fitness, parasitic female survival and fecundity. In contrast, the concentration of IL4 produced by MLN and concentration of RMCPII in intestinal tissue were separately associated with parasite survival and fecundity, respectively. These results are consistent with the well-documented observation that Th2-type responses correlate with protection against nematode infection in experimental infections [2]. Th2-type cytokines also play a key role in the resistance of different human age-classes to *Trichuris trichiura* and *Ascaris lumbricoides* infection [28]–[30] concomitant with convex age-intensity profiles for these infections. The concentration of IgA, IgG_1_ and IgG_2a_ changed during an infection; the concentration of IgA increased to a peak and then declined, whereas the concentration of IgG_1_ and IgG_2a_ both increased steadily to a plateau; and the concentration of IgE remained relatively unchanged (Figure 3). These results contrast, for example, with a study of childhood *T. trichiura* infections where parasite-specific IgG_1–4_ increased to a peak in 8–11 year old cohorts and declined by age 12–15, whereas IgE increased steadily [31].

The individual immune parameters measured showed correlated and concerted responses within this experiment (Figure 3 and Table 9). The concentration of two cytokine markers of a Th2-type immune response, IL4 and IL13, produced by parasite antigen-stimulated MLNs were strongly correlated to each other. The concentration of anti-*S. ratti*-IgG_1_ and of RMCPII were also positively correlated. Principal component analysis indicated that PCA1 correlated positively to all the immune measures analysed (Table 10 and Figure 4), with the exception of IgE, whereas PCA2 had two negatively correlated groups of immune parameters, MLN IL4, IL13 and IFNγ, and IgG_1_ and RMCPII. PCA1 may therefore reflect general immune activation following infection (Figure 5), whereas PCA2 reflects temporal separation between cytokine mediators versus immunoglobulin and mast cell effectors.

The experimental and statistical approach that we describe here goes some way towards interpreting the correlated suite of immune responses elicited by experimental nematode infection in the context of age-intensity profiles found in natural field infections [26]. Further work in this area could investigate, either in field or laboratory infections, the immunodynamics of concomitant multiple-species (or multiple-strain) infections acquired in trickle infections, which are likely to be the norm in the field [32]. Another priority is the explicit incorporation of within-host immunological dynamics, parameterised from experimental and field data, into population-level epidemiological models of nematode transmission, prevalence, intensity and disease [33].
